# Performance analysis of data resampling on class imbalance and classification techniques on multi-omics data for cancer classification

**DOI:** 10.1371/journal.pone.0293607

**Published:** 2024-02-29

**Authors:** Yuting Yang, Golrokh Mirzaei

**Affiliations:** 1 Department of Computer Science and Engineering, The Ohio State University, Columbus, Ohio, United States of America; 2 Department of Computer Science and Engineering, The Ohio State University, Marion, Ohio, United States of America; Islamia University of Bahawalpur: The Islamia University of Bahawalpur Pakistan, PAKISTAN

## Abstract

Cancer, in any of its forms, remains a significant public health concern worldwide. Advances in early detection and treatment could lead to a decline in the overall death rate from cancer in recent decades. Therefore, tumor prediction and classification play an important role in fighting cancer. This study built computational models for a joint analysis of RNA seq, copy number variation (CNV), and DNA methylation to classify normal and tumor samples across liver cancer, breast cancer, and colon adenocarcinoma from The Cancer Genome Atlas (TCGA) dataset. Total of 18 machine learning methods were evaluated based on the AUC, precision, recall, and F-measure. Besides, five techniques were compared to ameliorate problems of class imbalance in the cancer datasets. Synthetic Minority Oversampling Technique (SMOTE) demonstrated the best performance. The results indicate that the model applying Stochastic Gradient Descent (SGD) for learning binary class SVM with hinge loss has the highest classification results on liver cancer and breast cancer datasets, with accuracy over 99% and AUC greater than or equal to 0.999. For colon adenocarcinoma dataset, both SGD and Sequential Minimal Optimization (SMO) that implements John Platt’s sequential minimal optimization algorithm for training a support vector machine shows an outstanding classification performance with accuracy of 100%, AUC, precision, recall, and F-measure all at 1.000.

## Introduction

Cancer has been the leading cause of death in United States. According to the American Cancer Society, over 1.9 million new cancer cases are projected to be diagnosed in the US in 2023, and over 609,000 people are expected to die from the disease [1]. The number of projected new cancer cases and deaths underscores the urgency to explore advanced techniques for improved cancer classification. As a heterogeneous disease comprised of multiple subtypes, the prognosis and prediction at an early stage is a critical aspect in the cancer treatment. With the advancement of technology, machine learning has emerged as a powerful tool in discovering information from the surge of complex medical data.

Over the past two decades, a diverse range of machine learning techniques and feature selection algorithms have found widespread application in disease prognosis and prediction [2–4]. Uddin et al. provided a survey of different supervised machine learning techniques for disease prediction [5]. Mirzaei et al. provided reviews of machine learning techniques for detection of Alzheimer’s disease [6, 7]. Vardhan et al. [13] compared different machine learning techniques including Decision Tree, Naïve Bayes, SVM, Random Forest, and Logistic Regression to predict heart diseases [8]. Kourou et al. reviewed the machine learning techniques used in cancer prognosis and prediction [9]. However, the lack of external validation to assess the predictive performance of the models remains a common hindrance in various machine learning classification studies, leading to a low rate of penetration into clinical practice.

Human cancers undergo complex rearrangements at the genetic, transcriptional, and proteomic levels, driving the process of oncogenesis [10]. Developments in omics technologies have facilitated the progress in understanding this disease. Omics is field of molecular biology that involves characterizing and quantifying the genome, transcriptome, and proteome of a biological sample to study its structure, function, and dynamics. Therefore, the pursuit of a holistic view of cancer behavior and the discovery of novel therapeutic vulnerabilities have led to an increasing interest in multi-omics analysis within cancer research over the past decades.

In addition, burgeoning medical science and technology enables the collection of vast amounts of multi-omics data. The Cancer Genome Atlas (TCGA) [11] is one such landmark cancer dataset. The TCGA project established molecular basis for more than 33 cancer types with over 20,000 primary cancer and matched normal samples, generating comprehensive molecular profiles of genomic, epigenomic, transcriptomic, and proteomic data. The complex datasets support an integrated framework of analysis that involves data from RNA seq, microRNA expression, protein expression, copy number variation (CNV), and DNA methylation.

The goal of this paper is to explore the application of different machine learning techniques for the classification of tumor and normal samples from various cancer types based on a global landscape of RNA seq, CNV, and DNA methylation. RNA seq provides insight into the transcriptome of a cell [10]. CNV is a particular form of structural variation that is involved in genomic analysis, which examines DNA sequences and aims to comprehend the correlations between diseases and genomic alterations [10]. DNA methylation is a fundamental aspect of epigenomics, which explores the epigenome’s chemical modifications and protein alterations that regulate gene expression without altering the underlying nucleotide sequence [12].

Our study’s significance lies in its comprehensive evaluation of multiple machine learning methods, tailored to liver cancer, breast cancer, and colon adenocarcinoma. It aims to highlight performance differences among classifiers for these specific cancer types and offers a thorough assessment of each method. We undertook classifications between normal and tumor instances in 403 samples of liver cancer, 841 samples of breast cancer, and 295 samples of colon adenocarcinoma from TCGA dataset separately using 18 machine learning methods. By exploring the distinct performances of classifiers on these cancer types, we shed light on their specific characteristics and potential for personalized treatment approaches.

In addition, our study recognizes the varying importance of different metrics at distinct stages of diagnosis. For instance, a classifier’s sensitivity (recall) holds significant value during early-stage screening [13]. By considering multiple metrics, including accuracy, AUC, precision, recall, and F-measure, our approach enhances the robustness of our analysis and affords a comprehensive evaluation of the classifiers’ performance across various diagnostic phases. This multifaceted assessment aids in providing more nuanced insights and a well-rounded understanding of the classifier’s effectiveness in different clinical contexts.

Notably, we address the challenge of the *curse of dimensionality* in the multi-omics dataset, a particular challenge for the use of early concatenation in multi-omics integration, characterized by having a vast number of features while the number of available data points is low [14]. To mitigate this, we employed principal component analysis (PCA) as a feature extraction and dimension reduction technique. Additionally, the dataset has a limited number of normal samples. Therefore, we evaluate various resampling techniques to reduce bias stemming from imbalanced class distributions, enhancing the efficiency and performance of the machine learning methods in handling high-dimensional data.

## Methods

### Data preprocessing

Data from TCGA were downloaded using R package TCGA-Assembler and include RNA seq, copy number, and DNA methylation. The dataset for breast, liver, and colon cancers were retrieved from The Cancer Genome Atlas Liver Hepatocellular Carcinoma Collection (TCGA-LIHC), Breast Invasive Carcinoma Collection (TCGA-BRCA), and Colon Adenocarcinoma Collection (TCGA-COAD), respectively. The data were first preprocessed in R language to combine normal and tumor samples into an integrated table for samples that have matching records for three omics (RNA seq, DNA methylation, and CNV). The combined dataset for LIHC contains 54105 features for each of 403 samples, among which 39 samples are normal and 364 samples are tumors. BRCA dataset contains 54096 features for each of 841 samples, among which 65 samples are normal and 776 samples are tumors. The COAD dataset contains 19 normal samples and 276 tumor samples with each of them having 54129 features. Principal Component Analysis (PCA) [15] was performed as part of data preprocessing to reduce the dimensionality of the data. Also, different techniques were performed to overcome the class imbalance issue for normal data. Classifications were done on Waikato Environment for Knowledge Analysis (WEKA) [16], a Java-based software. WEKA provides a collection of built-in machine learning algorithms for data mining tasks with user-friendly interfaces. It also includes tools for data pre-processing and visualization. A ten-fold cross-validation was used to evaluate the performance of each kind of machine learning algorithms and prevent overfitting. Lastly, to address the imbalance between positive and negative samples, various resampling techniques, including Synthetic Minority Oversampling Technique (SMOTE), Random Under-Sampling, Cost-Sensitive Training (CST), NearMiss, and Tomek Links, were compared.

### Dimensionality reduction

Principal component analysis (PCA) is a technique that has been widely applied in machine learning and data analysis for dimension reduction [15]. By linear transformation, the original features can be converted into principal components, which is a new set of orthogonal features. PCA aims to generates a smaller amount of artificial set of attributes that can retain most of dataset’s information, so it is particularly useful for the case of high dimensional data. The process of dimensionality reduction includes normalizing the data, calculating the covariance matrix that shows the relationships between features, and getting the eigenvectors and eigenvalues of the covariance matrix to calculate principal components and the amount of variation explained by each principal component. In this study, we used “prcomp” [17] function in R to perform PCA on the original data. As part of the preprocessing, normal samples were considered negative and labeled 0; tumor samples were considered positive and labeled 1. The features with value 0 were removed, due to raising error when calling prcomp() for PCA as they cannot be rescaled to unit variance during normalization. Normalization is performed to ensure that the variables are on the same scale and avoid the situation that the resulting principal components are skewed by scales. Number of the features before and after PCA and minimum number of principal components to explain 95% of total variance for each dataset is shown in Table 1.

**Table 1:** Number of original features (before PCA), and number of features after dimensionality reduction using PCA.

Cancer Type	# of original features	# of the features after dimensionality reduction
LIHC	54105	403
BRCA	54096	841
COAD	54129	295

### Class imbalance

Class imbalance refers to the case in which the distribution of classes in a dataset is not uniform, resulting biased machine learning model that cannot be able to accurately identify instances of the minority class and misleading accuracy. The situation that the number of tumor samples significantly outnumbers that of normal samples was observed in all three datasets (LIHC, BRCA, and COAD). Of samples included in the study, about only 9.7% of samples in the LIHC, 7.7% of samples in BRCA, and 6.4% of samples in COAD were considered as normal (non-tumor) samples. The classifiers tend to bias towards the positive class that has a greater number of samples. Therefore, five different techniques were applied to treat the imbalanced classes including four resampling methods and Cost Sensitive Training, as described in the following.

#### Synthetic Minority Oversampling Technique (SMOTE)

This is a popular data augmentation technique that works by generating synthetic instances for the minority class [18]. SMOTE randomly selects a minority class instance and its *k* nearest neighbors (*k*NN) of the same class and then interpolates data points on the join lines between the selected instances. In this study, we set the value of *k* to 5 for the *k*NN algorithm. The number of minority class samples was doubled after applying SMOTE once.

#### Random Under-Sampling (RUS)

This is a technique to address the class imbalance by randomly removing examples from the majority class in the training data [19]. In this study, it involves screening out a subset of tumor samples from the cancer dataset at random to ensure the tumor and normal class of an equal number. The feature of this technique is simple and easy to implement. However, random under-sampling can lead to the loss of potentially valuable information from the majority class, especially when the class imbalance is severe.

#### Informed Under-Sampling (IUS)

Another type of under-sampling we applied are guided by certain criterions, such as Tomek Links [20] and NearMiss [19]. Compared to random under-sampling, informed under-sampling tends to keep more information in the original dataset. Tomek links are defined as the pairs of instances of opposite classes that are closet to each other in feature space and under-sampling is done by removing the instance of the majority class in those pairs. Similarly, NearMiss refers to a collection of under-sampling methods that remove the instances of the majority class based on their distances to the instances of the minority class, and to remove the rest. There are three kinds of NearMiss, each follows different standard of selecting majority class instances. In this study, we applied NearMiss-1 [21] to keep only the majority class examples with minimum average distance (Euclidean distance) to the minority class examples.

#### Cost-Sensitive Training (CST)

This technique is a machine learning approach that takes into account the misclassification costs associated with different classes during model training [22, 23]. CST allows the model to assign higher importance to minority classes with fewer examples and thus helps to improve the model’s performance on these under-represented classes. We employed CST training with penalized learning algorithm that increases the cost of misclassifying the minority class to alleviate the bias resulting from data imbalance [24].

SMOTE, RUS, and CST were performed by applying WEKA’s SMOTE filter, SpeadSubsample filter, and CostSensitiveClassifier, respectively. NearMiss and Tomek Links were performed using the ‘themis’ [25] package in R language, which provides extra recipes steps for dealing with unbalanced data.

## Modeling and evaluation metrics

Overall, 18 machine learning algorithms were selected to build classifiers, including Bayes Net [26], Naïve Bayes [27], Logistics [28], Stochastic Gradient Descent (SGD for binary class SVM) [29, 30], Simple Logistics [31], Sequential Minimal Optimization (SMO) [32–34], Voted Perceptron [35], K-nearest Neighbors (IBk) [36], Decision Table [37], JRip [38], OneR [39], PART [40], Decision Stump [41], J48 [42], Logistic Model Trees (LMT) [31, 43], Random Forest [44], Random Tree [45], and REP Tree [46]. Different classification models were evaluated to identify the classifier with high performance in terms of accuracy, AUC, precision, recall, and F-measure. The five metrics were used to measure the overall quality of the model’s predictions for binary classification problems. The performance metrics are described as follows:

**Accuracy** is calculated as the ratio of the number of correct predictions to the total number of predictions made, as:

$$Accuracy=\frac{TP+TN}{TP+TN+FP+FN}$$ (1)

where TP, TN, FP, FN represent true positive, true negative, false positive, and false negative, respectively.

**Precision** measures how many of the positive predictions were correct. It is the proportion of true positives out of the total predicted positives, representing the classifier’s ability to correctly identify positive instances.


$$Precision=\frac{TP}{TP+FP}$$ (2)

**Recall**, also known as True Positive Rate (TPR) or Sensitivity, measures the proportion of actual positive cases that were correctly identified. It is calculated as the ratio of true positives to the sum of true positives and false negatives, as:

$$Recall=\frac{TP}{TP+FN}$$ (3)

**F-measure**, also known as the F1 score, is the harmonic mean of precision and recall. It is a single value that balances both precision and recall, and is commonly used when both false positives and false negatives are equally important.


$$F1=\frac{2}{\frac{1}{Precision}+\frac{1}{Recall}}=\frac{2\times Precision\times Recall}{Precision+Recall}$$ (4)

**AUC** (Area under ROC curve) is a measure of the classifier’s power in distinguishing between positive and negative instances. It represents the area under the Receiver Operating Characteristic (ROC) curve, which plots the true positive rate against the false positive rate.

## Results and discussion

Results of this study are presented in two parts. In the first part, we report the effects of adjusting the balance of classes of SMOTE, RUS, CST, IUS (NearMiss, Tomek Links) by observing the results of classifiers’ performance on the data after mitigating the class imbalance and comparing the accuracy, AUC, precision, recall, and F-measure of the classification on LIHC dataset and verifying on BRCA dataset. The class imbalance method with most significant effect was selected and applied on COAD dataset. In the second part, the results show the comparisons among the classifiers built on different machine learning methods on BRCA, COAD, and LIHC datasets.

### Effect of class imbalance techniques

Tables 2–6 show the effects of different class imbalance techniques (SMOTE, RUS, CST, IUS -NearMiss, Tomek Links) on different classifiers. Table 2 shows resulted classification accuracy. It can be seen that the highest classification rate is obtained from SMOTE+SGD with 99.5% accuracy rate. SMOTE also provides relatively higher accuracy rate with most of the classifiers compared to the other class imbalance techniques. Tomek Links provides the next highest accuracy with three classifiers. Tomek Links+Simple Logistics, Tomek Links+SMO, and Tomek Links+LMT provides 98% classification rate. Also, CST + Simple Logistics provides 98.7% accuracy rate. Table 3 shows the AUC of the combination of different class imbalance techniques with classifiers. It can be seen that SMOTE+Logistics provides the highest AUC of 1, next Tomek Links+Simple Logistics, Tomek Links+LMT, and CST+Simple Logistics provides AUC of 0.99. Tables 4 and 5 show the precision and recall of the techniques, and Table 6 shows the F1 score. The highest F1 score of 0.99 is achieved from SMOTE, CST, and Tomek Links. However, SMOTE provides this outstanding performance with SGD, Simple Logistics, SMO, and LMT, while CST provides this high F1 score with Simple Logistic, and also Tomek Links provides this high F1 score with Simple Logistics and SMO. Overall, SMOTE provide higher performance with more classifiers compared than the other class imbalance techniques, although Tomek Links has just relatively less performance than SMOTE but also similar to SMOTE it works along with more classifiers.

**Table 2:** Comparison of the classification accuracy of different class imbalance models with classifiers on LIHC dataset.

Class Imbalance Classifier	SMOTE	RUS	CST	IUS (NearMiss)	IUS (Tomek Links)
Bayes Net	96.38%	91.02%	96.02%	92.30%	96.01%
Naïve Bayes	95.47%	89.74%	94.54%	91.02%	94.73%
Logistics	97.96%	71.79%	88.08%	56.41%	77.44%
SGD	**99.5%** ^**a**^	84.61%	91.56%	82.05%	97.24%
Simple Logistics	98.41%	92.30%	**98.75%**	96.15%	**98.49%**
SMO	99.32%	91.02%	98.01%	84.61%	**98.49%**
Voted Perceptron	64.02%	75.64%	64.51%	79.48%	64.16%
IBk (Nearest Neighbor)	17.64%	50%	36.47%	51.28%	40.35%
Decision Table	92.98%	92.30%	96.77%	89.74%	93.48%
JRip	97.51%	93.58%	97.27%	89.74%	97.74%
OneR	91.40%	93.58%	93.30%	82.05%	93.98%
PART	98.19%	97.43%	97.51%	96.15%	97.49%
Decision Stump	90.04%	93.58%	90.32%	91.02%	89.22%
J48	96.83%	97.43%	97.76%	96.15%	96.74%
LMT	98.41%	92.30%	96.27%	96.15%	**98.49%**
Random Forest	97.96%	93.58%	91.31%	93.58%	96.99%
Random Tree	92.08%	80.76%	92.55%	73.07%	91.72%
REP Tree	96.60%	92.30%	96.52%	91.02%	97.99%

^a^Highest accuracy.

**Table 3:** Comparison of the AUC of different class imbalance models with classifiers on LIHC dataset.

Class Imbalance Classifier	SMOTE	RUS	CST	NearMiss	Tomek Links
Bayes Net	0.97	0.95	0.97	0.96	0.96
Naïve Bayes	0.96	0.94	0.95	0.93	0.95
Logistics	**1.00** ^**a**^	0.93	0.079	0.85	0.11
SGD	0.99	0.84	0.56	0.82	0.86
Simple Logistics	0.99	0.98	**0.99**	0.96	**0.99**
SMO	0.99	0.91	0.92	0.84	0.93
Voted Perceptron	0.98	0.92	0.96	0.97	0.98
IBk (Nearest Neighbor)	0.49	0.50	0.56	0.5	0.59
Decision Table	0.95	0.95	0.96	0.92	0.93
JRip	0.95	0.93	0.90	0.93	0.88
OneR	0.81	0.93	0.73	0.82	0.94
PART	0.96	0.97	0.86	0.94	0.92
Decision Stump	0.89	0.89	0.87	0.86	0.89
J48	0.95	0.96	0.91	0.94	0.92
LMT	0.99	0.98	0.99	0.96	**0.99**
Random Forest	0.99	0.98	0.97	0.97	0.98
Random Tree	0.86	0.80	0.76	0.73	0.76
REP Tree	0.92	0.90	0.87	0.91	0.93

^a^Highest AUC.

**Table 4:** Comparison of precision of different class imbalance models with classifiers on LIHC dataset.

Class Imbalance Classifier	SMOTE	RUS	CST	NearMiss	Tomek Links
Bayes Net	0.99	0.90	0.98	0.92	0.99
Naïve Bayes	0.99	0.89	0.98	0.90	0.99
Logistics	**1.00** ^**a**^	0.94	0.90	0.72	0.90
SGD	**1.00** ^**a**^	0.96	0.91	**1.00** ^**a**^	0.97
Simple Logistics	**1.00** ^**a**^	1.00	0.99	**1.00** ^**a**^	0.99
SMO	0.99	0.97	0.98	**1.00** ^**a**^	0.98
Voted Perceptron	1.00	0.95	**1.00** ^**a**^	**1.00** ^**a**^	1.00
IBk (Nearest Neighbor)	N/A	N/A	0.93	**1.00**	0.94
Decision Table	0.96	0.94	0.97	0.89	0.95
JRip	0.98	0.92	0.97	0.87	0.98
OneR	0.92	0.92	0.94	0.83	0.94
PART	0.98	1.00	0.98	0.97	0.98
Decision Stump	0.98	0.92	0.90	0.97	0.93
J48	0.98	1.00	0.98	0.97	0.98
LMT	**1.00**	1.00	0.96	1.00	0.99
Random Forest	0.98	0.92	0.91	0.91	0.97
Random Tree	0.95	0.77	0.95	0.75	0.95
REP Tree	0.97	0.92	0.97	0.92	0.98

^a^Highest precision.

**Table 5:** Comparison of recall of different class imbalance models with classifiers on LIHC dataset.

Class Imbalance Classifier	SMOTE	RUS	CST	NearMiss	Tomek Links
Bayes Net	0.96	0.92	0.96	0.92	0.96
Naïve Bayes	0.95	0.89	0.95	0.92	0.95
Logistics	0.97	0.46	0.97	0.20	0.84
SGD	**0.99**	0.71	**1.00**	0.64	**0.99**
Simple Logistics	0.98	0.84	0.99	0.92	0.98
SMO	0.99	0.84	0.99	0.69	0.99
Voted Perceptron	0.56	0.53	0.60	0.59	0.60
IBk (Nearest Neighbor)	0.00	0.00	0.31	0.02	0.36
Decision Table	0.95	0.89	**0.99**	0.89	0.97
JRip	0.98	0.94	**0.99**	0.92	**0.99**
OneR	0.97	0.94	0.98	0.79	**0.99**
PART	0.99	0.94	0.98	0.94	0.98
Decision Stump	0.89	0.94	**1.00** ^**a**^	0.84	0.95
J48	0.97	0.94	0.98	0.94	0.98
LMT	0.98	0.84	0.99	0.92	0.98
Random Forest	**0.99**	0.94	**1.00**	**1.00**	**0.99**
Random Tree	0.95	0.87	0.96	0.69	0.95
REP Tree	0.98	0.92	0.986	0.89	**0.99**

^a^Highest recall.

**Table 6:** Comparison of F1 score of different class imbalance models with classifiers on LIHC dataset.

Class Imbalance Classifier	SMOTE	RUS	CST	NearMiss	Tomek Links
Bayes Net	0.97	0.91	0.97	0.92	0.97
Naïve Bayes	0.97	0.89	0.96	0.91	0.97
Logistics	0.98	0.62	0.93	0.32	0.87
SGD	**0.99** ^**a**^	0.82	0.95	0.78	0.98
Simple Logistics	**0.99** ^a^	0.91	**0.99** ^**a**^	0.96	**0.99** ^**a**^
SMO	**0.99** ^**a**^	0.90	0.98	0.81	**0.99** ^**a**^
Voted Perceptron	0.72	0.68	0.75	0.74	0.75
IBk (Nearest Neighbor)	N/A	N/A	0.47	0.05	0.52
Decision Table	0.95	0.92	0.98	0.89	0.96
JRip	0.98	0.93	0.98	0.90	0.98
OneR	0.94	0.93	0.96	0.81	0.96
PART	0.98	0.97	0.98	0.96	0.98
Decision Stump	0.93	0.93	0.94	0.90	0.94
J48	0.98	0.97	0.98	0.96	0.98
LMT	**0.99**	0.91	0.98	0.96	0.99
Random Forest	0.98	0.93	0.95	0.95	0.98
Random Tree	0.95	0.81	0.95	0.72	0.95
REP Tree	0.98	0.92	0.98	0.90	0.98

^a^Highest F1 score.

By applying the SMOTE filter, the number of normal samples in LIHC dataset was doubled from 39 to 78. Since SMOTE newly generated a certain amount of synthetic normal samples that are sequentially added to the original dataset, this is likely to lead to overfitting and poor generalization performance. To overcome this limitation, the order of samples passed through the models was randomly shuffled using Randomize [47] filter in the WEKA. Randomizing the order of samples passed through the classifier can help to reduce the impact of any ordering-related biases in the training data.

After applying SMOTE, improvements in the AUC were observed in 14 classifiers, however the AUC of Simple Logistics and LMT did not change. Especially for Logistics (a multinomial logistic regression model with a ridge estimator), its accuracy increased approximately 20%. While for models of Voted Perceptron and IBk the AUC dropped. However, the overall improvement outweighs the negative influence on the performance of Voted Perceptron and IBk because the performances of these two classifiers were already relatively poor before resampling data. As shown in Tables 2 and 3, SMOT increased the classification accuracy of 12 classifiers to ≥ 95% and AUC of 13 classifiers to ≥ 0.95. SGD shows the highest accuracy of 99.5%, with the AUC at 0.99, the highest precision at 1.00, the highest recall at 0.99, and the highest F-measure at 0.99. SMO also shows an accuracy over 99% with the highest recall. It is also worth noting that Logistics produced the highest AUC at 1.00.

Through the SpreadSubsample filter with the distributionSpread being set to 1.0, WEKA produced a random subsample of the original dataset with the same number of samples for both classes. As a result, Random Under-Sampling decreased the number of positive samples to 39. However, 325 instances were wasted, and the overall performance of classifiers dropped except the model of PART. The accuracies of 12 classifiers have dropped after Random Sub-Sampling. The effect of Random Sub-Sampling is useful in PART, Decision Stump, and J48, resulting in the increase of both accuracy and AUC. However, the highest accuracy generated by PART can only reach 97.4% (Table 2). As shown in Tables 2 and 3, after Random Sub-Sampling, only 2 classifiers’ accuracy reaches 95% and 7 classifiers’ AUC are greater than 0.95, which is less effective compared to SMOTE. Simple Logistics and LMT have the highest AUC at 0.98, though their performance were weakened by the preprocessing of Random Under-Sampling.

CostSensitiveClassifier is built-in metaclassifier model that makes its base classifier cost sensitive in WEKA. It was used to realize the penalized training to account for imbalanced classes by assigning different costs to different types of misclassifications. The cost matrix was a 2 by 2 matrix with default costs of false positive (FP) and false negative (FN) at 1.0. Since the dataset lacks negative instances, the classifiers tend to skew towards the majority class of positive. Therefore, to improve the classification performance on normal instances, the cost of detecting FP was raised from 1.0 to 2.0. After CST, only four classifiers’ AUC were improved. Nine classifiers’ accuracy reached 95% and seven classifier’s AUC were greater than 0.95 (Tables 2 and 3). Simple Logistics has the highest accuracy at 98.7% and the highest AUC at 0.99, but it was not improved by applying CST. Overall, CST is less effective than SMOTE.

In addition to Random Under-Sampling, two informed under-sampling techniques, NearMiss-1 and Tomek Links, were also examined on LIHC datasets using the package ‘themis’ [25] in R language. NearMiss-1 retains the closest majority class sample to each minority class instance and removed 325 tumor samples. Similar to Random Under-Sampling, the overall performances of the classification models were significantly reduced. After NearMiss-1, Random Forest shows the highest AUC at 0.97 (Table 3). Only four classifier’s Percent Correctly Classified reach 95% and five classifiers have AUC over 0.95 (Tables 2 and 3).

In LIHC dataset, there are only two Tomek Links (the pairs of instances of opposite classes that are closet to each other in feature space). Tomek Links only removed major class samples from two pairs of positive-negative instance bindings, generating a dataset that is still imbalanced with 39 normal and 362 tumor instances. After Tomek links, Simple Logistics shows the highest accuracy at 98.50% and the highest AUC at 0.99 (Tables 2 and 3). However, compared to other techniques, it is not obvious that the classifiers’ performance has changed between the data before and after the application of Tomek Links.

As summarized in Table 7, among the five techniques, the highest accuracy, AUC, and F1 score can be achieved on LIHC data by applying SMOTE. The results also indicate that SMOTE works along with more classifiers. Overall performance of classifiers on SMOTE is better than the other techniques in all three cancer types. In addition, of the 18 models which we selected, there are 12 classifiers reach the highest accuracy (>95%), 13 classifiers reach the highest AUC (>0.95), and 14 classifiers reach the highest F1 score (>0.95) on the data after SMOTE on LIHC dataset.

**Table 7:** Summary of the five techniques’ effect on addressing class imbalance and resulting classification performance on LIHC dataset.

Technique	Highest AUC	Highest Accuracy	Highest F1 score	# Classifiers with accuracy > 95%	# Classifiers with AUC > 0.95	# Classifiers with F1 > 0.95
SMOTE	1.00	99.54%	0.99	12	13	14
RUS	0.98	97.43%	0.97	2	7	2
CST	0.99	98.75%	0.99	9	7	14
NearMiss-1	0.97	96.15%	0.96	4	5	5
Tomek Links	0.99	98.49%	0.99	11	6	14

To verify the generality of using SMOTE as the techniques to address class imbalance, the same series of techniques were also conducted on the BRCA dataset. We found that while BRCA dataset has the largest number of samples, no Tomek Link was detected so applying Tomek Links cannot change the number of samples. As summarized in Table 8, taking the results on the datasets after Tomek Links as the benchmark, SMOTE was the only technique that improves # Classifiers with accuracy > 95% and # Classifiers with AUC > 0.95. In addition, SMOTE boosts the highest accuracy that the classifiers can achieve. Although RUS and NearMiss-1 result in AUC at 1.0, but the results cannot prove a perfect classifier because the great loss of sample sizes impaired overall quality of classifications in terms of accuracy and F1 score. A classifier can have a perfect AUC but low accuracy and F1 score when it is which means the classifiers it is particularly sensitive to the true positive rate (TPR) while disregarding the true negative rate (TNR). This means that the classifier excels at correctly classifying positive instances, leading to a high AUC value as it effectively separates positive and negative instances. However, the classifier also has a relatively high false positive rate (FPR) as it may incorrectly classify some negative instances as positive, leading to a low precision.

**Table 8:** Summary of the five techniques’ effect on addressing class imbalance and resulting classification performance on BRCA dataset.

Technique	Highest AUC	Highest Accuracy	Highest F1 score	# Classifiers with accuracy > 95%	# Classifiers with AUC > 0.95	# Classifiers with F1 > 0.95
**SMOTE**	0.99	99.66%	0.99	9	9	11
**RUS**	1.00	96.92%	0.96	2	5	2
**CST**	0.99	98.81%	0.99	7	5	14
**NearMiss-1**	1.00	97.69%	0.97	2	5	2
**Tomek Links**	0.99	98.81%	0.99	7	5	12

To sum up, taking the balance between positive and negative samples and the classifiers’ performance into consideration, SMOTE was selected to make up for the lack of normal samples in BRCA and COAD datasets. A summary of resulting datasets is shown in Table 9.

**Table 9:** Summary of SMOTE’s effect on different cancer types.

Cancer Type		Number and Percentage of Normal Instances		Number and Percentage of Tumor Instances	
LIHC	Original	39	9.7%	364	90.3%
	After SMOTE	78	17.6%	364	82.4%
BRCA	Original	65	7.7%	776	92.3%
	After SMOTE	130	14.3%	776	85.7%
COAD	Original	19	6.4%	276	93.6%
	After SMOTE	38	12.1%	276	87.9%

### Tumor detection for different cancer types

In the following section, we present the performance of various shallow machine learning models on the binary classification of the multi-omics data between normal and cancer patients, with regards to their accuracy, AUC, precision, recall, and F-measure for LIHC (Table 10), BRCA (Table 11), and COAD (Table 12). Table 10 is a pooling of results of SMOTE on LIHC data from Tables 2–7. This table shows the results of applying different machine learning algorithms on LIHC dataset. In general, the machine learning-based models exhibited comparable performance, but there were a few methods where this was not the case. SGD showed the highest accuracy of 99.54%. with a remarkable AUC at 0.99. SGD also demonstrates the highest precision at 1.0, the highest recall at 0.99, and the highest F score at 0.99, indicating its nearly perfect capability to correctly classify true positives while minimizing false positives and false negatives. SMO also shows an excellent result of classification accuracy over 99% with the highest recall and second highest F score. It is worth noting that Logistics had the highest AUC at 1.0 but AUC at 97.96% meaning that the model has perfect discrimination between the positive and negative classes, but the current threshold used by accuracy cannot achieved a perfect classification. Five models generated precision at 1, representing 100% of positive predictions in the dataset were correct.

**Table 10:** Comparison of the performance metrics of different classifiers for LIHC.

Classification Algorithms	Accuracy (%)	AUC	Precision	Recall	F-measure
Bayes Net	96.38%	0.97	0.99	0.96	0.97
Naïve Bayes	95.47%	0.96	0.99	0.95	0.97
Logistics	97.96%	**1.00**	**1.00**	0.97	0.98
SGD	**99.54%**	0.99	**1.00**	**0.99**	**0.99**
Simple Logistics	98.41%	0.99	**1.00**	0.98	0.99
SMO	99.32%	0.99	0.99	**0.99**	0.99
Voted Perceptron	64.02%	0.98	**1.00**	0.56	0.72
IBk (Nearest Neighbor)	17.64%	0.49	N/A	0.00	N/A
Decision Table	92.98%	0.95	0.96	0.95	0.95
JRip	97.51%	0.95	0.98	0.98	0.98
OneR	91.40%	0.81	0.92	0.97	0.94
PART	98.19%	0.96	0.98	0.99	0.98
Decision Stump	90.04%	0.89	0.98	0.89	0.93
J48	96.83%	0.95	0.98	0.97	0.98
LMT	98.41%	0.99	**1.00**	0.98	0.99
Random Forest	97.96%	0.99	0.98	0.99	0.98
Random Tree	92.08%	0.86	0.95	0.95	0.95
REP Tree	96.60%	0.92	0.97	0.98	0.98

**Table 11:** Comparison of the performance metrics of different classifiers for BRCA.

Classification Algorithms	Accuracy (%)	AUC	Precision	Recall	F-measure
Bayes Net	90.72%	0.97	0.99	0.89	0.94
Naïve Bayes	88.96%	0.94	0.99	0.88	0.93
Logistics	95.80%	0.99	0.99	0.95	0.97
SGD	**99.66%**	0.99	**1.00**	0.99	**0.99**
Simple Logistics	99.11%	**0.99**	0.99	0.99	0.99
SMO	**99.66%**	0.99	0.99	0.99	**0.99**
Voted Perceptron	66.55%	0.99	**1.00**	0.61	0.75
IBk (Nearest Neighbor)	14.34%	0.50	N/A	0	N/A
Decision Table	84.87%	0.93	0.97	0.84	0.90
JRip	96.57%	0.93	0.98	0.97	0.98
OneR	90.28%	0.73	0.92	0.97	0.94
PART	97.35%	0.94	0.98	0.98	0.98
Decision Stump	85.65%	0.85	0.85	**1.00**	0.92
J48	94.70%	0.90	0.97	0.96	0.96
LMT	99.11%	**0.99**	0.99	0.99	0.99
Random Forest	97.35%	0.99	0.97	0.99	0.98
Random Tree	92.16%	0.81	0.94	0.96	0.95
REP Tree	96.02%	0.95	0.97	0.97	0.97

**Table 12:** Comparison of the performance metrics of different classifiers for COAD.

Classification Algorithms	Accuracy (%)	AUC	Precision	Recall	F-measure
Bayes Net	99.04%	0.97	0.98	1.00	0.99
Naïve Bayes	98.08%	0.97	0.99	0.98	0.98
Logistics	91.71%	**1.00**	**1.00**	0.90	0.95
SGD	**100%**	**1.00**	**1.00**	**1.00**	**1.00**
Simple Logistics	99.36%	**1.00**	**1.00**	0.99	0.99
SMO	**100%**	**1.00**	**1.00**	**1.00**	**1.00**
Voted Perceptron	53.18%	**1.00**	**1.00**	0.46	0.63
IBk (Nearest Neighbor)	12.10%	0.49	N/A	0.00	N/A
Decision Table	94.58%	0.95	0.96	0.97	0.96
JRip	97.77%	0.92	0.97	1.00	0.98
OneR	95.22%	0.85	0.96	0.98	0.97
PART	97.45%	0.93	0.97	0.99	0.98
Decision Stump	95.85%	0.86	0.97	0.982	0.97
J48	96.17%	0.90	0.97	0.986	0.97
LMT	99.36%	**1.00**	**1.00**	0.993	0.99
Random Forest	97.77%	0.99	0.97	**1.000**	0.98
Random Tree	92.03%	0.85	0.96	0.942	0.95
REP Tree	96.49%	0.89	0.97	0.986	0.98

For BRCA dataset, several models demonstrated desirable performance. SGD and SMO have the highest accuracy at 99.66% with both of their AUC greater than or equal to 0.99 and both have the highest F score at 0.99, among which SGD generates precision at 1. Besides, both Simple Logistics and LMT have the highest AUC at 0.99 and accuracy over 99%, showing their good power in the classification of normal and tumor instances. Different from the liver cancer’s results, Decision Stump has the highest recall at 1 meaning 100% actual positive instances are correctly predicted (Table 11).

The classification results of COAD are presented in Table 12. Compared to other two types of cancers, the machine learning based models tend to have better overall performance on COAD dataset. SGD and SMO show their perfect performance to classify the normal and tumor instances with 100% accuracy and AUC, precision, recall, and F score all 1.0. Six methods including Logistics, SGD, Simple Logistics, SMO, Voted Perceptron, and LMT have both AUC and precision at 1. Bayes Net, SGD, SMO, JRip, and Random Forest achieved recall of 1.

Nevertheless, classifiers’ performance on the COAD dataset raises the concern about the higher predictive value and its potential association with its smaller size. To address that, we conducted additional analysis and extended our discussion on this matter. While it is true that smaller datasets can sometimes lead to overfitting issues, we took precaution to mitigate this possibility. Firstly, we employed rigorous cross-validation techniques to ensure the generalizability of our models and avoid overfitting. Secondly, we evaluated the difference in results on datasets of varying sizes. Our findings suggest that while the COAD dataset’s smaller size may have influenced its predictive value, the overall trends of classifiers’ performance observed in our analysis remain consistent across datasets. However, we acknowledge that sample size remains an important consideration in machine learning studies, and we have included the influence of sample numbers in the limitations.

For tumor detection based on multi-omics data in the three cancer datasets, the model using SGD drastically outperformed all the other tested machine learning algorithms with nearly perfect classification performance. In the COAD dataset, both SGD and SMO are able to produce the best classification result. The model based on SMO also demonstrates performance surpassing the other methods in the datasets of the other two cancer types, so it can be considered as the second-best classifier. Moreover, it is observed in all the datasets that some methods are not suitable for the tumor detection based on multi-omics data for their exceptionally low scores in classification performance, such as Voted Perceptron and Nearest Neighbor. Nevertheless, Voted Perceptron has satisfying AUC and precision, though its accuracy and recall are very low, meaning that the proportion of correctly identified positive cases out of all actual positive cases in the dataset is low. Voted Perceptron’s high AUC with low accuracy suggests that the model has a severe imbalanced classification issue so that the default threshold resulted in poor performance. In this case, Voted Perceptron’s biased towards predicting negative instances, which leads to a high specificity (true negative rate) but a low sensitivity (true positive rate) at the chosen threshold value. Therefore, the threshold needs to be fine-tuned to achieve optimal classification performance.

As summarized in Table 13, we defined the best classifier was defined as the one that achieved the highest score on the accuracy, AUC, precision, recall, and F-measure the most times. Among the 18 models selected, SGD provided the best performance in all three datasets and SMO is also the best classifier in COAD dataset. Both SGD and SMO demonstrated the prominent classification performance in all three datasets, with accuracy over 99%, AUC, precision, recall, and F-measure all over 0.99. For COAD dataset, the overall performance of classifiers is higher than their performance on the dataset of LIHC and BRCA. SGD and SMO even achieved perfect classification in COAD dataset.

**Table 13:** Summary of the classification performance on the datasets of different cancer types.

Cancer Type	Highest Accuracy (%)	Highest AUC	Highest Precision	Highest Recall	Highest F-Score	Best Classifier(s)
LIHC	99.54%	1.00	1.00	0.99	0.99	SGD
BRCA	99.66%	0.99	1.00	1.00	0.99	SGD
COAD	100%	1.00	1.00	1.00	1.00	SGD, SMO

## Limitation

One potential limitation of our study may stem from the omics data we considered. Although the cancer data we used including genomics, epigenomics, and transcriptomics provide valuable insights into the molecular landscape of the disease, we acknowledge that multi-omics analysis could be further enhanced by integrating proteomic data, which would provide a more comprehensive view of cancer biology. Compared to the genome or transcriptome, proteome exhibits a closer association with cellular phenotypes compared to the genome or transcriptome [48]. In addition, recent progress in mass spectrometry technology allows for high-throughput proteomic analysis in large-scale cancer studies [49, 50].

Another limitation is the influence of sample numbers on the obtained results. While our analysis showcased impressive classification performance on the chosen datasets, and we employed 10-fold cross-validation to mitigate overfitting, it is crucial to account for the potential influence of varying sample sizes. This is especially relevant when utilizing PCA for the dimensionality reduction of high-dimensional multi-omics dataset, as the dataset’s variability is distributed in a manner where each data point significantly contributes to the variance along its respective principal component. Consequently, this can impact the classification outcomes and cause possibility of overfitting. The COAD dataset, with its relatively smaller size, exhibited particularly high performance levels for certain classifiers. However, the implications of this outcome might be influenced by the limited number of samples in the dataset. Variability in sample size can introduce bias and affect the generalizability of the results to larger and more diverse populations. Additionally, as sample size can influence the ability to detect true positive and negative cases, the potential for overfitting in smaller datasets must also be carefully addressed. Therefore, the conclusions drawn from our study should be interpreted in the context of sample size considerations and further investigations with larger and more balanced datasets are warranted to validate the robustness of our findings.

Additionally, in data preprocessing, there exist defects in the resampling techniques or how the techniques were applied. First, we only applied SMOTE once, which can only double the number of minority class, resulting in restricted effects of tackling class imbalance issue. This restriction can be improved by SMOTE multiple times in future studies. Second, under-sampling methods such as Random Under-Sampling and NearMiss had very limited effects in addressing class imbalance because of their fatal deficiency that many majority class instances are ignored. But this problem can be overcome by some possible solutions [51]. Future studies could be conducted with more balanced data or ratio to mock cancer incidence rate of population to reduce the impact of class imbalance and improve the validity of results.

Lastly, the building of classification models on WEKA could be a possible limitation of this study. WEKA’s graphical user interface (GUI) allows users to perform machine learning tasks with ease, but the values of all parameters of the classification models used in this study were remained the default. For example, only Nearest Neighbor (k = 1) was examined by applying IBk classifier in WEKA, but IBk also supports K-Nearest Neighbors algorithm by setting parameter. Therefore, using classifier with default parameters might not be able to realize the optimal result. Similarly, the limitation of default parameters also lies in the data preprocessing filters applied by this study. The parameters of the filters to address class imbalance of the datasets need to be further finetuned to verify that it exerts ideal effect. For instance, the value of FP in the cost matrix of the CST Classifier was only intuitively increased by double, but it may not be the best cost matrix to solve the imbalance of the datasets we used.

## Conclusion

This study aims at the classification between the primary cancer and normal samples of different types of cancer including LIHC, BRCA, and COAD. Data are obtained from TCGA dataset based on multi-omics data of samples’ molecular characteristics including RNA Seq, CNV, and DNA methylation, trying to find the best classifier for each kind of cancer dataset. Five techniques were tested on LIHC dataset to address the class imbalance in the primary dataset which among SMOTE is most effective in reducing the effect of class imbalance and improving the performance of classifiers. Multiple shallow classification models were examined on the datasets of LIHC. BRCA, and COAD after being processed by SMOTE, to classify the tumor and normal samples. Among the chosen set of 18 models, SGD consistently displayed the most superior performance across all three datasets, while SMO emerged as the top classifier for the COAD dataset. Both SGD and SMO exhibited remarkable classification capabilities across all three datasets, demonstrating accuracy rates exceeding 99%, along with AUC, precision, recall, and F-measure values surpassing 0.99, showcasing the substantial potential of these two classifiers in the realm of cancer diagnosis utilizing multi-omics data.
